# Plot based heritability estimates and categorization of cassava genotype response to cassava brown streak disease

**DOI:** 10.1016/j.cropro.2018.02.008

**Published:** 2018-06

**Authors:** A. Okul Valentor, M. Ochwo-Ssemakula, T. Kaweesi, A. Ozimati, E. Mrema, E.S. Mwale, P. Gibson, E. Achola, R. Edema, Y. Baguma, R. Kawuki

**Affiliations:** a National Crops Resources Research Institute, Root Crops Program, P.O. Box 7084, Kampala, Uganda; b Makerere University, Department of Agricultural Production, P.O. Box 7062, Kampala, Uganda

**Keywords:** Cassava, Cassava brown streak disease, Categorization, Heritability, Resistance

## Abstract

Cassava brown streak disease (CBSD) caused by *Cassava brown streak virus* (CBSV) and *Ugandan cassava brown streak virus* (UCBSV) is a threat to food security in sub-Saharan Africa, where the disease persistently reduces overall root quality and quantity resulting in up to 100% yield losses. Complexities in CBSD symptom expression and the damage caused on leaves, stems and roots throughout the 12 months of cassava growth require that appropriate ways of categorizing genotype response and optimal stages of evaluation be identified. This study aimed at: 1) determining plot based heritability of CBSD based on symptom expression and 2) categorizing genotype resistance to CBSD based on symptom expression. Herein, 41 genotypes were evaluated for two years at Namulonge with an additional evaluation conducted across three locations. Evaluations were done at three, six, nine and twelve months after planting. Genotype responses to CBSD varied significantly. High broad sense heritability estimates of up to 0.81 (incidence) and 0.71 (severity) were obtained.

Average disease severity scores had higher broad sense heritability estimates (0.53 and 0.65) than maximum disease severity scores (0.33 and 0.61) for root and foliar severities respectively. These findings are important in choosing an appropriate evaluation method for CBSD. Genotypes displayed differing CBSD responses in type, locality and severity of symptoms. This suggested that genotypes had differences in mechanisms of resistance that can be exploited in CBSD resistance breeding.

## Highlights

• Heritability of CBSD resistance is higher when evaluations are done on the basis of average severity scores per plot.
• Cassava genotypes displayed differing CBSD responses in the type, locality and severity; testifying different mechanisms of resistance.
• Correlation analyses between foliar and roots were largely negative, indicating different genetic control in the plant parts.

## 1 Introduction

Cassava (*Manihot esculenta* Crantz.) is affected by cassava brown streak disease, one of the seven most serious threats to food security in the world (Pennisi, 2010). The disease is caused by two genetically distinct virus species, CBSV and UCBSV (family, *Potyviridae*: genus, *Ipomovirus*) (Mbanzibwa et al., 2009a, Mbanzibwa et al., 2009b, Winter et al., 2010). The most recent study has shown that, in addition to the two species (CBSV and UCBSV), three clades within UCBSV exist, indicating the possibility of four distinct species of CBSD causative viruses (Ndunguru et al., 2015). These viruses are transmitted by the whitefly *Bemisia tabaci* as a vector (Maruthi et al., 2005, Mware et al., 2009). These two factors, variability in the causal agents and high populations of the vector are major challenges breeding programs are striving to check, particularly, in eastern and southern Africa, where the disease has so far caused huge losses (Legg et al., 2014).

Since the first report of CBSD in 1936 in Tanzania, the disease has been endemic to cassava growing areas of Kenya and lakeshore areas of Malawi (Nichols, 1950). In recent years, CBSD has spread to northern Mozambique, Uganda, Burundi and Rwanda, where it is threatening cassava production and food security (Hillocks et al., 2002, Alicai et al., 2007, Ntawuruhunga and Legg, 2007). Further spread and occurrence of CBSD has also been confirmed in Burundi (Bigirimana et al., 2011) and eastern Democratic Republic of Congo (DRC) (Mulimbi et al., 2012), with the most recent outbreaks reported as far as Gabon and Angola (FAO, 2013). To mitigate any further spread of the disease, several options have been suggested; phytosanitation, clean seed systems, quarantine and breeding for resistance. The most effective options include; breeding for resistance and implementation of clean seed systems (Legg et al., 2014, Mcquaid et al., 2015).

However, the development of CBSD resistant varieties requires understanding of the genetics and inheritance of resistance to the disease and identification of new sources of resistance. Breeding for CBSD resistance was initiated at Amani Research Station, Tanzania in 1930s (Storey, 1936). Since then, resistance and/or tolerance to the disease constitute a major breeding objective for breeding programmes in eastern and southern Africa, where the disease is widespread. Other breeding programmes have demonstrated that genetic gains are a function of: a) selection accuracy, b) selection intensity, c) additive genetic variance, and d) cycle time. Gains in CBSD breeding can, thus, only be attained through optimization of these factors.

A few genetic studies on CBSD have been conducted in Mozambique (Zacarias and Labuschagne, 2010), Kenya (Munga, 2008), Uganda (Tumuhimbise et al., 2014) and Tanzania (Kulembeka et al., 2012). Most of these studies have reported the relative importance of GCA effects and, hence, additive effects for CBSD resistance (Kulembeka et al., 2012, Munga, 2008, Tumuhimbise et al., 2014). Contrary findings were only observed in Mozambique (Zacarias and Labuschagne, 2010). Kawuki et al. (2016) identified clones with higher levels of tolerance to CBSD The authors also provided further insights into CBSD genetics through identification of genomic regions associated with resistance. The urgent need for optimizing CBSD evaluations was also highlighted. This study, therefore, aimed at quantifying broad sense heritability (H^2^) associated with CBSD evaluations in clonal populations of cassava at different plant growth stages.

The nature and extent of damage caused by CBSD in leaves, stems and roots throughout the 12 months maturity period of cassava requires that thresholds i.e., optimal stages of evaluations be identified. This will enable proper ranking of cassava genotypes under evaluation, which is particularly relevant for early selection stages (i.e., seedling and/or clonal) where several genotypes are evaluated. Variability in patterns of symptom expression within different cassava genotypes complicates selection of tolerant or resistant genotypes. According to Hillocks et al. (2002) and Rwegasira et al. (2012a), some cassava genotypes show both foliar and root symptoms while others show either foliar or root symptoms with varying severity levels. Earlier reports also showed that foliar symptoms for CBSD were more clearly expressed on leaves than on stems (Hillocks and Jennings, 2003, Rwegasira et al., 2012b). It has, however, been noted that there is variation in foliar symptom expression, with some genotypes showing leaf symptoms, but no observable disease on the stem or vice versa. This study, therefore contributed to developing a stem severity evaluation scale (other than the routinely used scale that combines both leaf and stem), which is a modification of the stem severity scale used by Rwegasira et al. (2012b).

Symptom expression on a host plant is an index of host-pathogen interaction and is as such used to infer the level of resistance of a given genotype to that particular pathogen. The differences observed in CBSD symptom expression in different plant parts with time creates a need to develop a universal approach of estimating levels of resistance based on symptom expression. For this reason the current study also focused on categorizing genotype resistance to CBSD based on symptom expression.

## 2 Materials and methods

### 2.1 Genetic materials

Forty one (41) diverse cassava genotypes (Table 1) that had earlier been evaluated for key agronomic traits at Namulonge (central Uganda) were selected from the training population and used for this study. The training population comprised 429 clones that are part of the Next Generation Cassava Breeding Project that is exploring the usefulness of genomic selection (www.cassavabase.org) for cassava genetic improvement (Wolfe et al., 2016).

**Table 1:** Pedigree of 41 cassava genotypes evaluated for response to CBSD.

Clone	Female Parent	Male Parent	Source
UG120001	TMS30572	MH95/0414	Full sib of IITA clones
UG120002	NASE 11	TMS 60142	Full sib of IITA clones
UG120006	TMS30572	MH95/0414	Full sib of IITA clones
UG120022	MM96/4271	Namikonga	Full sib of IITA clone x TZ clone-Namikonga
UG120024	MM96/4271	Namikonga	Full sib of IITA clone x TZ clone-Namikonga
UG120037	MM96/4271	Namikonga	Full sib of IITA clone x TZ clone-Namikonga
UG120048	TME 14	Namikonga	Full sib of IITA clone x TZ clone-Namikonga
UG120072	TME 204	MH95/0414	Full sib of IITA clones
UG120089	TMS30572	MH95/0414	Full sib of IITA clones
UG120099	I92/0067	MH95/0414	Full sib of IITA clones
UG120109	OO40	OO40	Selfed progeny of IITA clone
UG120113	MM96/4271	MH04/2588	Full sib of IITA clones
UG120135	MM96/4271	MH04/2575	Full sib of IITA clones
UG120146	CR5A-1	CR5A-1	Selfed progeny of CIAT CR-line
UG120154	CR5A-1	CR5A-1	Selfed progeny of CIAT CR-line
UG120156	Introduction TZ		Selection from TZ Seed Introduction-2005
UG120157	Introduction TZ		Selection from TZ Seed Introduction-2005
UG120160	CR21-6		Half Sib of CIAT CR-Line
UG120170	CR24-8		Half Sib of CIAT CR-Line
UG120172	CR24-8		Half Sib of CIAT CR-Line
UG120178	Introduction TZ		Selection from TZ Seed Introduction-2005
UG120189	Introduction TZ		Selection from TZ Seed Introduction-2005
UG120190	Introduction TZ		Selection from TZ Seed Introduction-2005
UG120192	Introduction TZ		Selection from TZ Seed Introduction-2005
UG120194	Introduction TZ		Selection from TZ Seed Introduction-2005
UG120221	Namukono	CR54-1	Full Sib of CIAT CR-Line x Ugandan local
UG120227	Njule red		Half sib of Ugandan local
UG120286	Kibao	CR36-2	Full Sib of CIAT CR-Line x Ugandan local
UG130001	TZ 140		Half Sib of TZ Material
UG130003	Unknown		Unknown
UG130006	TZ 140		Half Sib of TZ Material
UG130007	Unknown		Unknown
UG130010	TZ 140		Half Sib of TZ Material
UG130018	Unknown		Unknown
UG130033	Unknown		Unknown
UG130068	Unknown		Unknown
UG130083	Unknown		Unknown
UG130089	TME 204		Half sib of IITA clone
UG130098	Unknown		Unknown
NASE 14*			
TME 204*			

Note: IITA = International Institute of Tropical Agriculture; CIAT = International Center for Tropical Agriculture; TZ = Tanzania; CBSD and agronomic data of the test clones can be accessed from cassavabase (www.cassavabase.org). *Checks: NASE 14 and TME 204, which are respectively classified as resistant and susceptible to CBSD (Kaweesi et al., 2014).

### 2.2 CBSD field evaluations

Initially, these 41 genotypes were evaluated in the field for response to CBSD at a single site, Namulonge which is characterized by high CBSD pressure and high whitefly populations (Abaca et al., 2012, Kaweesi et al., 2014, Pariyo et al., 2015), for two consecutive years (2013 and 2014). During each year, trials were established using incomplete block designs with two replications. Each clone was represented by 10 plants in a single row. Spreader rows of TME 204, a highly susceptible variety (Kaweesi et al., 2016), were planted after every five rows to augment CBSD disease pressure. Visual assessment for CBSD symptom expression on foliage was done for all plants in a plot on the basis of maximum severity score obtained per plot (maximum severity score). A third CBSD field re-evaluation was undertaken in 2015 at three locations [Namulonge, Kamuli (eastern Uganda) and Kasese (western Uganda)] using un-replicated single row plots of 10 plants per row. CBSD susceptible (TME 204) and tolerant (NASE 14) genotypes were included as checks for comparison purposes.

Visual assessment for CBSD symptom expression on foliage and/or stems was done on both the average severity score of all individual plants assessed in a plot (average severity) and maximum severity score of a plot, at three, six and nine months after planting (MAP). Thus, the field evaluations were done consecutively for three years. It suffices to note that seedlings that formed the cassava training population were cloned in 2012; it's from these seedlings that stem cuttings were obtained for 2013 trial. Thereafter, planting materials were recycled for the 2014 and 2015 trials.

Foliar severity (degree of infection on each plant) was scored on a 1–5 scale, where 1 = no symptoms; 2 = mild symptoms (1–10%); 3 = pronounced chlorotic mottle and mild stem lesion (11–25%); 4 = severe chlorotic mottle and stem lesions (26–50%) and 5 = very severe symptoms (>50%). Stem severity was scored as follows; 1 = no stem symptoms; 2 = mild stem lesions (1–10%); 3 = pronounced stem lesions (11–25%); 4 = severe stem lesions and streaks (26–50%); 5 = very severe stem lesions and streaks, withering and die-back (>50%) (Gondwe et al., 2002, Rwegasira et al., 2012a).

At harvest, 12 MAP, all plants in a plot were uprooted and all roots individually assessed for CBSD necrosis. This was done using the 1–5 scale, where 1 = no necrosis; 2 = mild necrotic lesions (1–10%); 3 = pronounced necrotic lesions (11–25%); 4 = severe necrotic lesions (26–50%) with mild root constriction and 5 = very severe necrotic lesions (>50%) with severe root constrictions (Gondwe et al., 2002).

### 2.3 Data analysis

CBSD incidence on foliage, stem and roots for each plot was quantified as a ratio of number of plants and/or roots showing CBSD symptoms to total number of plants and/or roots harvested per plot. Data on disease incidence and severity were fitted to linear mixed models using lmer function built in lme4 package in R.

Three separate analyses were undertaken. First, the trials conducted at a single location (Namulonge) for the two seasons, 2013 and 2014; these datasets are referred to as dataset one. Secondly, the trial that was undertaken once in 2015 across three locations; this data set is referred to as dataset two. Thirdly, analysis done across seasons and locations, when common traits were measured in dataset one and two and those combined datasets are referred to as dataset three.

Dataset one was analyzed as RCBD (randomized complete block design). The following model was used: ***y***_***ij***_ = ***μ***+C_i_+***β***_j_ + ***e***_***ij***_, where ***y***_***ij***_ = plot measurement, ***μ*** = grand mean; C_i_ = clone effect; ***β***_j_ = effect of the replication; and ***e***_***ij***_ = residual. For dataset two, analysis was based on single row plots with locations considered as replications, with the following model: ***y***_***ij***_ = ***μ***+***E***_***j***_ + ***C***_i_ + ***e***_***ij***_, where ***y***_***ij***_ = plot measurement; ***μ*** = grand mean; ***E***_***j***_ = location effect; ***C***_i_ = clone effect and ***e***_***i***j_ = residual.

Data set three; across seasons and locations, analysis was done based on randomized complete block design (RCBD). The following model was used: ***y***_***ijk*1**_ = ***μ***+***β***_j_/(Sx***E***)_kl_ + C_i_ + S_k_ + ***E***_l_ + C***E***_il_ + CS_ik_ + C***E***S_ilk_ + ***e***_***ijk***1_, where ***y***_***ijk***_ = plot measurement; ***μ*** = grand mean; ***β***_j_/(Sx***E***)_kl_ = effect of the replication within “season x location”; C_i_ = clone effect; S_k_ = season effect; ***E***_l_ = location effect; C***E***_i1_ = effect of “clone x location”; CS_ik_ = effect of the “clone x season”; C***E***S_ilk_ = effect of “clone x location x season”;***e***_***ij***k_ = residual. Further, F-test for significance of treatments effects and computations of broad sense heritability estimates from variance components were done. Similarly, Pearson's correlation coefficients for the different traits were estimated from the combined datasets (n = 38), using *cor* function in R (R Core Team, 2013).

## 3 Results

### 3.1 CBSD field screening trials

Datasets associated with CBSD foliar, stem and root incidences and/or severity are presented in Table 2. At three MAP, there were significant differences (*p* ≤ 0.001) among genotypes for both foliar incidence and maximum foliar severity; average foliar severity also differed significantly among the genotypes (Table 2). No significant differences were observed for stem incidence, average stem severity and maximum stem severity at three MAP. Location effects were significant for foliar incidence, maximum foliar severity, stem incidence and maximum stem severity.

**Table 2:** Mean squares associated with CBSD foliar, stem and root severities and incidences at different crop growth stages.

Dataset 2					Mean squares			
Three MAP	SOV	D.f	3CBSDfi	3CBSDfm	3CBSDfs	3CBSDsi	3CBSDsm	3CBSDss
	Genotype	40	2554.70***	0.51**	1.12***	732.4	0.15	0.53
	Location	2	4263.20**	0.52	2.21**	3351.60*	0.52	2.19**
	Residual	55	834.6	0.23	0.4	762.1	0.13	0.4
	CV		73.65	31.92	35.31	184.63	30.67	45.36
	overall mean		41.47	1.53	1.89	14.66	1.19	1.43
	**H**^**2**^		**0.67**	**0.55**	**0.64**	**−0.04**	**0.13**	**0.25**
Six MAP	SOV	D.f	6CBSDfi	6CBSDfm	6CBSDfs	6CBSDsi	6CBSDsm	6CBSDss
	Genotype	40	2080.3***	0.85**	1529.78	1.63***	0.48	1.55**
	Location	2	3485.4**	1.27*	1876.58	0.42	0.48	0.22
	Residual	55	691.7	0.35	951.39	0.59	0.32	0.59
	CV		35.00	26.79	28.43	134.03	41.39	50.70
	overall mean		74.35	2.20	2.71	22.76	1.37	1.69
	**H**^2^		**0.67**	**0.59**	**0.38**	**0.64**	**0.33**	**0.62**
Nine MAP	SOV	D.f	9CBSDfi	9CBSDfm	9CBSDfs	9CBSDsi	9CBSDsm	9CBSDss
	Genotype	40	1321**	0.88***	1.4***	2451*	1.61**	2.43***
	Location	2	2717	1.09*	1.57*	5709*	6.87***	8.61***
	Residual	55	1145	0.31	0.55	1442	0.79	0.93
	CV		58.94	29.46	32.65	63.76	39.41	35.86
	overall mean		55.75	1.88	2.23	58.34	2.15	2.59
	**H**^2^		**0.13**	**0.65**	**0.61**	**0.41**	**0.51**	**0.62**
12 MAP	SOV	D.f	12CBSDrm	12CBSDrs	12CBSDri			
	Genotype	37	1.76**	3.22	1564.8*			
	Location	2	0.27	12.29**	1471.4			
	Residual	50	0.82	2.17	905.7			
	CV		53.61	50.01	92.02			
	Overall mean		1.62	2.83	30.12			
	**H**^2^		**0.53**	**0.33**	**0.42**			
c) Dataset 3	SOV	D.f	3CBSDfi	3CBSDfs	6CBSDfi	6CBSDfs	CBSDri	CBSDrs
	Genotype	40	3896.30***	1.40***	5006.20***	2.33***	2818.58***	4.55***
	Location	2	1681.40*	3.60***	3948.50**	6.66***	483.87	10.31***
	Season	2	9037.20***	0.99*	20807.50***	11.68***	2107.39**	20.70***
	Rep (Season x Location)	4	67.70	0.02	6353.60**	0.20	327.24	1.46
	Genotype x Location	60	970.20*	0.54**	894.10	0.76	683.70*	2.16**
	Genotype x Season	64	1093.40**	0.41	1116.00	0.44	913.13**	1.88*
	Genotype x Location x Season	64	1304.80	0.84	1554.60	0.06	1246.12	0.19
	Pooled error	58	526.60	0.27	743.00	0.30	1417.09	1.06
	**H**^2^		**0.69**	**0.71**	**0.81**	**0.45**	**0.69**	**0.32**

SOV = source of variation; D.f = degrees of freedom; 3, 6, 9 and 12 = three, six, nine and twelve MAP respectively; CBSDfs = cassava brown streak disease maximum foliar severity; CBSDss = cassava brown streak disease maximum stem severity; CBSDsm = cassava brown streak disease average stem severity; CBSDfm = cassava brown streak disease average foliar severity; CBSDfi = cassava brown streak disease foliar incidence; CBSDsi = cassava brown streak disease stem incidence; CBSDrm = cassava brown streak disease average root severity; CBSDrs = cassava brown streak disease maximum root severity; CBSDri = cassava brown streak disease root incidence; *, ** and *** represents significance at P < 0.05, 0.01, and 0.001, respectively; H^2^ = broad sense heritability. **Note:** Dataset 2 includes traits measured in 2015season only; dataset 3 are traits commonly measured in the seasons 2013, 2014and 2015.

At six MAP, highly significant differences (*p* ≤ 0.001) among genotypes for stem incidence and foliar incidence was observed (Table 2). Average foliar severity and maximum stem severity showed significant differences at *p* ≤ 0.01. Location effects were only significant for foliar incidence and average foliar severity (Table 2). At nine MAP, average foliar severity, maximum foliar severity and maximum stem severity showed highly significant differences (*p* ≤ 0.001) among genotypes; foliar incidence, average stem severity and stem incidence differed significantly among genotypes. It was only foliar incidence that was not significant for location effects (Table 2). At harvest, there were significant differences among genotypes for only root incidence and average root severity but not maximum root severity. Maximum root severity differed among locations (Table 2).

Significant differences were observed among seasons for foliar incidence (*p* ≤ 0.001) and maximum foliar severity (*p* ≤ 0.05) at three MAP; foliar incidence and maximum foliar severity were significant (*p* ≤ 0.001) at six MAP; significant differences in root incidence (*p* ≤ 0.01) and maximum root severity (*p* ≤ 0.001) were also observed (Table 2).

Significant “genotype x season” interactions were observed for maximum root severity (*p* ≤ 0.05), root incidence (*p* ≤ 0.01) and foliar incidence (*p* ≤ 0.01) at three MAP. The interactions between “genotype x location” were significant for root incidence (*p* ≤ 0.05), maximum root severity (*p* ≤ 0.01), foliar incidence (*p* ≤ 0.05), maximum foliar severity (*p* ≤ 0.01) at three MAP (Table 2). “Genotype x location x season” interactions were not significantly different for all CBSD traits measured (Table 2). Results further indicated that replications within “location x season” had a significant difference for only foliar incidence at six MAP at *p* ≤ 0.01 (Table 2).

### 3.2 Broad-sense heritability and correlations estimates

Data on plot-based heritability estimates are also presented in Table 2. At three MAP, maximum foliar severity had a slightly higher heritability estimate (0.64) than average foliar severity (0.55). The heritability estimate for foliar incidence was 0.67 (Table 2). At six MAP, average foliar severity had a higher heritability estimate (0.59) than maximum foliar severity (0.38).

A similar trend was observed at nine MAP, with average foliar severity indicating a higher heritability estimate (0.65) than maximum foliar severity (0.61). Heritability estimates for foliar incidences at six and nine MAP were 0.67 and 0.13 respectively (Table 2). Overall, heritability estimates for average foliar severity were higher than maximum severity.

For stem-based evaluations, heritability estimates at three MAP were generally low i.e. ≤ 0.25 (Table 2). At six MAP, heritability estimate for maximum stem severity was 0.62, while average stem severity was 0.33. A similar trend was observed at nine MAP with maximum stem severity indicating heritability estimates of 0.62, while average stem severity had heritability of 0.51 (Table 2). Stem incidences showed higher broad sense heritability estimates at six MAP (0.64) than at three and nine MAP. At harvest, average root severity showed a higher heritability estimate (0.53) than maximum root severity (0.33), while that of root incidence was 0.42 (Table 2).

Heritability estimates were generally higher when both seasons and locations were included in the analysis model. For example, at three MAP, heritability for foliar incidence and maximum foliar severity was 0.69 and 0.71 respectively. At six MAP foliar incidence and maximum foliar severity showed heritability estimates of 0.81 and 0.45 respectively. At harvest, heritability estimates of root incidence and maximum root severity was 0.69 and 0.32 respectively (Table 2).

Correlation analysis revealed a strong positive significant correlation between foliar incidence and average foliar severity r = 0.90, p ≤ 0.001; stem incidence and average stem severity r = 0.93, p ≤ 0.001; root incidence and average root severity 0.91, p ≤ 0.001. In addition, a positive significant correlation was revealed between average stem severity and average foliar severity r = 0.59, p ≤ 0.001; stem incidence and foliar incidence r = 0.44, p ≤ 0.01 (Table 3). The results further revealed that root necrosis severity exhibited a negative correlation with foliar (r = −0.01) and stem severity (r = −0.03). Similar trends were also observed between root, foliar and stem incidences (r = 0.01, −0.06) (Table 3).

**Table 3:** Phenotypic correlation between foliar, stem and root CBSD symptoms.

	CBSDfm	CBSDsm	CBSDrm	CBSDfi	CBSDsi	CBSDri	CBSDrs
CBSDfm	1.00						
CBSDsm	0.59***	1.00					
CBSDrm	−0.01	−0.03	1.00				
CBSDfi	0.90***	0.35*	−0.06	1.00			
CBSDsi	0.64***	0.93***	−0.07	0.44**	1.00		
CBSDri	−0.02	−0.15	0.91***	0.01	−0.15	1.00	
CBSDrs	−0.06	−0.04	0.73***	−0.02	−0.06	0.75***	1.00

CBSDsm = cassava brown streak disease average stem severity; CBSDfm = cassava brown streak disease average foliar severity; CBSDfi = cassava brown streak disease foliar incidence; CBSDsi = cassava brown streak disease stem incidence; CBSDrs = cassava brown streak disease maximum root severity; CBSDrm = cassava brown streak disease average root severity; CBSDri = cassava brown streak disease root incidence; *, ** and *** represents significance at P ≤ 0.05, 0.01, and 0.001, respectively; n = 38.

### 3.3 Categorizing CBSD resistance and/or tolerance levels based on symptoms

Based on the varied CBSD response in both incidence and severity, categorization of the genotypes was done. Overall, five categories were outlined, all dependent on the CBSD field symptom expression on leaves, stems and roots. The first category, comprised genotypes that had no foliar symptoms (UG120024 and UG120194) and/or those genotypes with very limited root necrosis as observed for genotypes UG120156 and UG120190 (Table 4).

**Table 4:** CBSD foliar, stem and root severities and incidences following field evaluations for three seasons in Uganda.

Response	Genotype	CBSDfs	CBSDfi	CBSDss	CBSDsi	CBSDrs	CBSDri
Category 1	UG120024	1.00	0.00	1.00	0.00	1.00	0.00
	UG120194	1.00	0.00	1.00	0.00	1.00	0.00
	UG120156	1.00	0.00	1.00	0.00	1.10	2.30
	UG120190	1.00	0.00	1.00	0.00	1.10	4.80
Category 2	UG120178	1.50	45.80	1.00	0.00	1.20	22.20
	UG120113	1.40	45.80	1.00	0.00	1.70	42.50
	UG120154	1.80	50.00	1.00	0.00	1.30	40.00
	UG130007	1.70	61.10	1.00	0.00	1.50	26.80
	UG120001	2.30	75.80	1.00	0.00	1.40	17.20
	UG120172	2.40	100.00	1.00	0.00	1.40	94.70
	UG120002	1.80	75.00	1.00	0.00		
	UG130098	1.80	83.30	1.00	0.00		
	UG120157	2.80	100.00	1.00	0.00		
Category 3	UG130010	2.40	96.30	1.00	0.00	1.00	0.00
	UG120037	2.30	100.00	1.00	0.00	1.00	0.00
	UG120189	2.00	55.60	1.40	22.20	1.00	0.00
	UG130006	2.50	100.00	1.50	45.80	1.00	0.00
Category 4	UG130089	1.70	55.60	1.10	6.70	1.40	40.60
	UG120072	2.20	88.00	1.40	7.40	1.40	9.00
	UG130003	2.00	75.00	1.80	50.00	1.50	28.60
	UG130018	2.10	69.30	1.60	36.70	1.70	31.80
	UG120048	2.20	91.70	1.30	26.70	2.00	56.90
	UG120089	2.20	89.60	1.10	13.30	1.70	51.00
	UG120109	2.20	81.90	1.10	4.80	1.30	14.80
	UG120146	2.20	84.70	1.40	25.00	2.70	54.00
	UG130001	2.20	92.10	1.20	22.20	2.00	55.60
	UG120286	2.40	93.80	1.20	12.50	2.00	34.60
	UG130033	2.30	75.00	2.40	75.00	1.70	16.70
	TME 204	2.40	79.20	1.30	22.60	4.70	98.90
Category 5	UG120192	3.00	100.00	3.00	100.00	1.20	12.50
	UG120160	2.50	100.00	1.30	25.00	2.00	38.50
	UG120227	3.20	95.80	1.10	4.20	1.30	28.40
	UG120135	2.70	66.70	1.80	44.40	2.00	49.00
	UG120170	2.50	100.00	1.40	35.70	1.10	21.40
	UG120022	2.70	95.80	1.60	49.40	1.20	12.90
	UG120006	2.80	100.00	1.80	50.00	1.70	24.20
	UG130068	3.10	100.00	1.70	57.10	1.70	46.90
	UG130083	2.90	90.00	2.00	33.30	1.70	20.30
	UG120099	2.90	100.00	1.60	60.00	2.50	66.30
	NASE 14	2.70	78.30	2.20	35.80	3.00	65.70
	Grand mean	2.20	74.35	1.37	18.21	1.62	30.12
	LSD 5%	1.19	52.60	1.13	61.65	3.00	61.48

CBSDss = cassava brown streak disease stem severity; CBSDfs = cassava brown streak disease foliar severity; CBSDfi = cassava brown streak disease foliar incidence; CBSDsi = cassava brown streak disease stem incidence; CBSDrs = cassava brown streak disease root severity; CBSDri = cassava brown streak disease root incidence; LSD = least significant difference. Data set based on evaluations conducted during 2013, 2014 and 2015 growing seasons.

The second category comprised genotypes UG120178,UG120154, UG130007, UG120001, UG120172, UG120002, UG130098, UG120157 and UG120113 that had no stem symptoms, but with moderate (incidence ≤45% and severities ≤2.4) or high (incidence ≥50% and severities ≥2.5) symptoms on leaves and in the roots. The third category is shown by UG130010, UG120037, UG120189 and UG130006. These genotypes have no root symptoms, but with no or few symptoms in the stem and moderate symptoms in the leaves (Table 4).

In the fourth category, are genotypes UG130089, UG120072, UG130003, UG130018, UG120048, UG120089, UG120109,UG120146, UG130001, UG120286, UG13003 and TME 204 that showed moderate symptoms in the leaves with few or moderate symptoms in the stem and the roots (Table 4). Category five included UG120192, UG120160, UG120227, UG120135, UG120170, UG120022, UG120006, UG130068, UG130083, UG120099 and NASE 14. These genotypes exhibited severe symptoms on leaves with severe and/or moderate symptoms on stems and roots (Table 4).

## 4 Discussion

The existence of CBSD menace for over 70 years on the continent, has, and continues to be a major challenge to farmers and scientists working towards its control. Variability in patterns of symptom expression has complicated the selection process despite the considerable time and resources invested. This study was thus undertaken to provide information on how to categorize response of genotypes to CBSD.

**Fig. 1:**
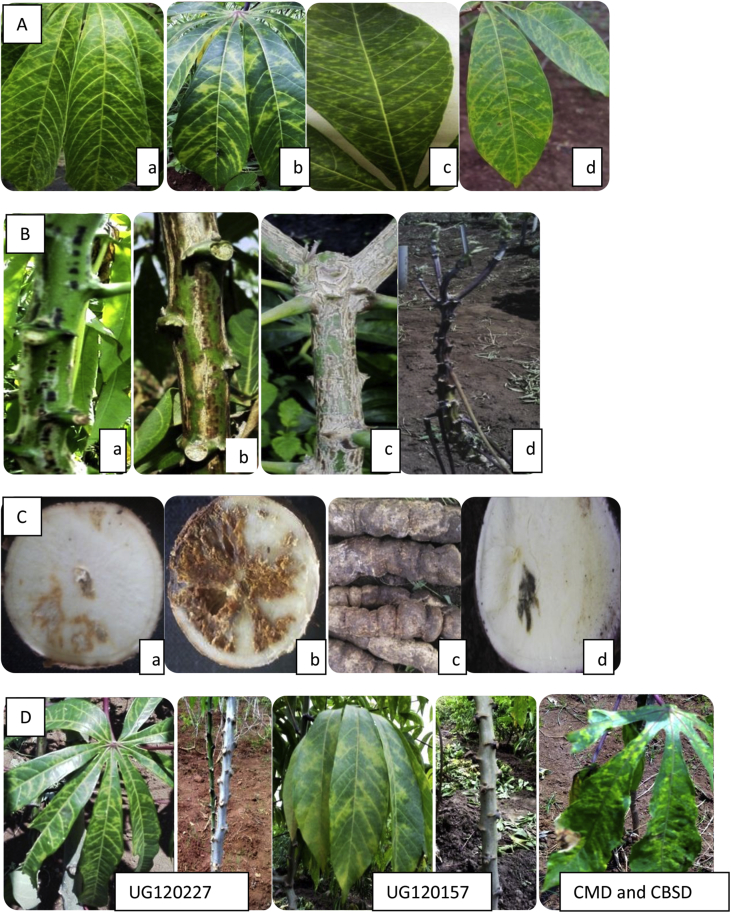
Varying cassava brown streak disease symptoms seen on leaves, stems and roots of infected cassava plants. A: Varied leaf symptoms a) leaf chlorosis, b) feathery patterns with chlorotic blotches along the margins of secondary, tertiary and main veins, c) mottling, and d) mixture of the symptoms. B: Varied stem symptoms a) purple lesions, b) brown lesions, and c) scaly brown lesions. C: Root symptoms showing a) chalky necrosis, b) brown necrosis, c) root constriction, and d) black necrosis. D: Genotype UG120227 and UG120157, associated with severe leaf symptoms with no stem symptoms. In some clones both CBSD and CMD symptoms appear on the plant. (For interpretation of the references to colour in this figure legend, the reader is referred to the Web version of this article.)

Varied CBSD responses were recorded among the tested genotypes (Table 4). There was also varied symptom types observed (Fig. 1), which could be attributed to the different causative virus species (Ndunguru et al., 2015). It was evident from the data that genotypes responded differently to CBSD (Table 2); an indication of the presence of genetic variability which favors selection of these genotypes for disease resistance. In addition, incidence and severity on different cassava genotypes varied with the growth stage of the plant (Table 2). For instance, foliar incidence had higher broad-sense heritability estimates at three and six MAP than at nine MAP. Stem incidence was highest at six MAP (Table 2). These results suggest that optimal evaluations of CBSD stem and foliar incidences can be done better at six MAP. Variability in CBSD incidence and severity among genotypes across locations implied that evaluation should be done in multiple environments.

Furthermore, some unique findings are highlighted on broad-sense heritability estimates with regards to severity. It was apparent that using average foliar and root severity scores, gives higher broad-sense heritability estimate than when maximum severity scores are used. For instance, in season 2015 it was observed that at six MAP, heritability for maximum foliar severity when compared to average foliar severity was 0.38 and 0.59 respectively.

A similar trend was observed between maximum root severity (0.33) and average root severity (0.53). This trend was further observed for foliar severity assessment at nine MAP. Nevertheless, a contrary trend was observed with stem severity, where maximum severity score had higher heritability estimates than average severity score at three, six and nine MAP (Table 2). As shown by Holland (2003), residual variance is composed of, variation due to plot effect and within plot variance. This implies that when average scores are used, the error within a plot is reduced, because using many plants per plot reduces the residual variance, which in-turn improves estimation of heritability. Similar results have been observed in cowpea where broad sense heritability on individual plant basis was 21.9%, while plant mean basis was 51.9% (Xu et al., 2009).

Across season and location heritability estimates were done for incidences, maximum foliar severity and root severity. As observed, these were generally higher at all growth stages (Table 2). These findings demonstrate that the magnitude of heritability of a given trait is not only affected by the type of genetic material, but also the environment (Ceccarelli, 1994, Hershey, 2012). This means that more than one season of evaluation is required to have effective selection for CBSD resistance.

Variations in broad-sense heritability across seasons have also been observed in CBSD traits evaluated in Mozambique; broad-sense heritability was 69.3% for the season 2004 and 48.0% for the season 2005 (Zacarias and Labuschagne, 2010). Seasons often have contrasting weather patterns and whitefly vector populations which could explain the difference.

Overall, it was evident that; i) there was a higher heritability for foliar severity, ii) high heritability for CBSD were obtained when evaluation were done on the basis of average severity score per plot as opposed to maximum severity score per plot, iii) at six MAP, foliar and stem incidences gave higher broad sense heritability estimates than at three and nine MAP, and iv) estimates of broad sense heritability varied with season. Therefore, heritability of resistance to CBSD could be improved greatly by conducting CBSD evaluation on the basis of average severity scores, to counteract the micro-environmental variations.

Regarding response of genotypes evaluated, varied symptom expressions were observed, suggestive of differential virus infection and consequently damage. The incidence and severity of shoot symptoms showed considerable variation, as has been seen in previous studies (Hillocks and Jennings, 2003, Rwegasira et al., 2012b, Kaweesi et al., 2014). For example, no leaf and/or stem symptoms were observed on UG120156, yet 100% incidence was observed in UG120192. In many cases a positive relationship was observed between shoot incidence and shoot severity. However, for genotypes UG120227 and UG120157, despite having severe symptoms in the leaves, exhibited no or very limited symptoms in the stem.

Reasons for this disparity remain unclear, although Kaweesi et al. (2014) reported the possibility of restriction of symptom expression as a mechanism of resistance. Expression of shoot symptoms with no or very limited root symptoms can be attractive, but should not be encouraged as this can lead to larger losses due to increased shoot symptoms resulting from high inoculum buildup up. Selection for reduced shoot incidence and symptom expression should, therefore, form an integral part of cassava breeding (Legg et al., 2014).

Normally, shoot evaluation for CBSD symptoms have been combining leaf and stem symptoms. As noted in this study, for some genotypes (e.g. UG120227), despite having severe leaf symptoms, stem symptoms were not observed (Fig. 1D). Such disparity creates a deviation from the normal scale and complicates evaluation. This would suggest that evaluation of stems and leaves could be done separately. Assessment of disease progress in all tested genotypes, except for asymptomatic ones, indicated that CBSD severity increased with time between three and six MAP. The intensity of foliar and stem symptoms (incidences and severity), thus, increased as the plants grew; a finding which concurs with other studies (Rwegasira et al., 2012a, Kaweesi et al., 2014). However at nine MAP an emergence of new asymptomatic leaves is a common phenomenon following defoliation of older leaves. Those young leaves can alter the true picture of foliar disease resistance making assessment at nine MAP challenging.

Root necrosis varied significantly as expected and was consistent with previous observations by Hillocks and Jennings (2003) and Kaweesi et al. (2014). Some genotypes had no root symptoms but with varying foliar and stem severity scores (Table 4). These genotypes are possible sources of CBSD resistance, once the virus load has been taken into account.

NASE 14 used as a resistant check in this study had severe root severity (3.0) and incidence (65.7%), with dieback. This could be attributed to degeneration due to the long period of exposure to the viruses; NASE 14 evaluations in 2015 coincided with its 10th year of exposure to CBSVs at Namulonge.

Correlation analyses between foliar, stem and roots severities and incidences provided varying genetic interpretations. For examples, high positive correlations were observed between foliar severity and incidence and between root severity and incidence. This finding suggests that in some cases foliar severity can be used to determine the extent of foliar incidence. On the contrary, root necrosis severity and incidence exhibited a negative correlation with foliar and stem severity and incidence, implying different genetic control. These findings are similar to those by Kaweesi et al. (2014). Moderate correlation between stem and foliar severity and incidence implies that a phenomenon like linkage or pleiotropy could be in play. This is an area that requires further investigation.

Also noted was the high influence of environment on expression of some CBSD traits. This observation could be attributed to genotype susceptibility levels, predominant virus species in locality and/or season, and climatic factors that either influences the abundance of whitefly vectors and/or the growth rate of the crop (Katono et al., 2015). The discovery of four distinct virus species (Ndunguru et al., 2015), is likely to further complicate the extent of genotype by environment interaction, as CBSD symptom expression associated with specific virus species are likely to differ between environments.

This therefore calls for a more precise phenotyping pipeline to have selection within location or stable genotypes across locations.

As observed by Nuwamanya et al. (2015), viral attack affects the accumulation of secondary metabolites within the host plant thereby inducing specific resistance mechanisms. This in turn causes an alteration in the plant metabolism which results into visible phenotypic and biochemical differences between diseased and healthy plants. This could partly explain the varied symptom expression and severities observed on leaves, stems and/or and roots (Fig. 1 and Table 4).

In a study by Kaweesi et al. (2014), even though NASE 14 was described to exhibit resistance to virus accumulation, few plants succumbed to infection by CBSVs and showed very high root severity (4 or 5) and incidence (90–100%), which was coupled with reduction in growth and in some cases dieback. In this study, NASE 14 had high virus titre; besides, most plants succumbed and showed high severity (3.0) and incidence (67.5%) on foliage and roots, while other genotypes remained asymptomatic on both foliage and roots. It can therefore be hypothesized that NASE 14 possesses partial resistance that breaks down under high inoculum pressure especially since stakes (stems) have been recycled for more than 10 years.

High virus accumulation over time increases disease susceptibility. A threshold, therefore, seems to exist at which the virus can overcome the plant defense mechanism thereby causing die back and necrosis in affected plants, as witnessed in NASE 14. Monitoring virus accumulation is, therefore, vital towards establishing the durability of resistance and in designing seed systems for cassava planting materials.

A number of definitions exist for virus resistance terminology (Thresh et al., 1998, Politowski and Browning, 1978). According to Thresh et al. (1998), truly resistant cultivars are not readily infected, even when exposed to large amounts of vector-borne inoculum. When infected, these cultivars develop inconspicuous symptoms that are not associated with obvious deleterious effects on growth and yield. They also support low virus titre and are, thus, poor sources of inoculum.

Resistance is, therefore, determined through virus titre and symptom expression. From this study, CBSD severity and incidence on leaves, stems and roots was measured, and thus, we limit our categorization of genotypes to disease symptom expression. The five categories created here are important as they help define responses in genotypes, and thus enabling selection. Based on field responses, a few genotypes notably, UG120024, UG120194, UG120156 and UG120190 were found to be associated with no and/or very limited CBSD symptoms, and can thus be considered as disease resistant. Accordingly, these genotypes are potential sources of CBSD resistance.
